# Heterogeneous expression of Lgr5 as a risk factor for focal invasion and distant metastasis of colorectal carcinoma

**DOI:** 10.18632/oncotarget.23144

**Published:** 2018-07-10

**Authors:** Zhong Zheng, Huiping Yu, Qin Huang, Hongyan Wu, Yao Fu, Jiong Shi, Ting Wang, Xiangshan Fan

**Affiliations:** ^1^ Department of Pathology, The Affiliated Drum Tower Hospital, Nanjing University Medical School, Nanjing, 210000, P.R. China; ^2^ Department of Pathology and Laboratory Medicine, Veterans Affairs Boston Healthcare System and Harvard Medical School, Boston, MA, USA

**Keywords:** colorectal cancer, Lgr5, heterogeneous expression, progression

## Abstract

Leucine-rich repeat-containing G-protein-coupled receptor 5 (Lgr5) is a downstream target gene of the Wnt/β-catenin signaling pathway and identified as a marker of cancer stem-like cells of colorectal carcinoma (CRC). Here, the heterogeneous expression pattern of Lgr5 and its clinical significance were studied by the method of immunohistochemistry in 204 CRC tumors at various pTNM stages. Lgr5 expression was found in 82.4% (168/204) cases, significantly more common in neoplastic cells at the infiltrative front (*n =* 59.5%, 110/185) or at the expanding front (*n =* 36.4%, 59/162) than at the tumor center (*n =* 16.7%, 34/204; *P* < 0.01). Tumor budding (TB) was discovered with significantly higher Lgr5 expression (*n =* 39.3%, 57/145, *P =* 0.03) and significantly positively correlated between Lgr5 expression and TB grade (*r* = 0.19, *P =* 0.02). Additionally, both positive Lgr5 expression and a high TB grade were significantly correlated to the depth of tumor invasion, lymph node metastasis, pTNM stage, and perineural invasion (*P* < 0.01). The study results suggest that heterogeneous expression of Lgr5 may be a risk factor for local invasion and distant metastasis of CRC.

## INTRODUCTION

Leucine-rich repeat-containing G-protein-coupled receptor 5 (Lgr5) is a member of seven-transmembrane GPCRs family identified in recent years. It is a downstream target gene of the Wnt/β-catenin signaling pathway and plays an important role in the embryonic development and organogenesis [1, 2]. Recently, some studies show that Lgr5 is a marker of stem cells in small intestine and colon [3] and cancer stem-like cells of colorectal carcinoma (CRC) [4], and its over-expression is also detectable in CRC. Although R-spondin has been confirmed as a ligand of Lgr5 [5], the intracellular signaling pathway related to Lgr5 is still unclear, and the correlation of Lgr5 with the occurrence, maintenance and metastasis of CRC is also poorly understood. Few studies have been conducted to investigate the role of Lgr5 in the occurrence and development of CRC. The results from our previous study showed that Lgr5 expression in neoplastic cells of CRC was significantly more often found at the invasive front than at the tumor center; high Lgr5 expression was also detected in the region of tumor budding (TB), residual cancer tissues surrounding the necrotic foci, and distant metastatic organs of 42 CRC cases staged as pTNM IV. These results indicated that Lgr5 over-expression was possibly related to proliferation, invasion, and prognosis of CRC [6]. To further investigate Lgr5 expression in CRC tumors at various stages, we in the present study aimed to analyze and compare the heterogeneous expression of Lgr5 at the tumour center, invasive front, and TB of CRC staged as pTNM I–IV and explore its relationship with clinicopathological and prognostic characteristics of CRC.

## RESULTS

There were 120 males and 84 females, and the median age was 64 years (range: 34–87). CRC, located at the left colon (splenic flexure of transverse colon, descending colon, sigmoid colon and rectum) was found in 146 patients and 58 in the right colon (ileocecal colon, ascending colon and hepatic flexure of transverse colon). In addition, pTNM stage I was found in 24 patients, stage II in 71, stage III in 98, and stage IV in 11. The concordance rate of Lgr5 expression and the evaluation of tumor budding was 93.6% and 92.2% respectively between two experienced pathologists. The minimal difference was resolved with consensus after a joint reading.

### Lgr5 expression in CRC tumors

Of 204 CRC tumors, 168 (82.4%) demonstrated Lgr5 expression that was significantly correlated to the depth of invasion, lymph node metastasis, pTNM stage, and perineural invasion (*P* < 0.01). Lgr5 expression was not significantly related to patient age, gender, tumor size, tumor location, tumor differentiation and lymphovascular invasion (*P* > 0.05) (Table 1). Thirty-six cases with negative expression of Lgr5 were confirmed with negative immunostaining in one additional tumor block of every CRC case.

**Table 1:** Lgr5 expression in CRC tissues and its relationship with clinicopathological characteristics of CRC from 204 patients

		Negative (lgr5)	Positive (lgr5)	X^2^	*P*	*r*	*P*
Gender	Male	23	97	0.46	0.50	0.01	0.85
	Female	13	71				
Age (years)	≤ 64	17	94	0.91	0.34	-0.04	0.55
	> 64	19	74				
Tumor size (cm) (average)	≤ 4.4	21	100	0.02	0.90	-0.14	0.04
	> 4.4	15	68				
Tumor location	Right-side	10	48	0.01	0.92	0.01	0.89
	Left-side	26	120				
Tumor grade	Low	32	139	0.83	0.36	0.08	0.26
	High	4	29				
pT	1	6	0	21.60	0.00	0.12	0.08
	2	6	23				
	3	20	130				
	4	4	15				
pN	0	25	73	8.12	0.02	0.09	0.22
	1	8	74				
	2	3	21				
pM	0	36	157	1.37	0.24	0.07	0.35
	1	0	11				
pTNM	1	11	13	15.67	0.00	0.19	0.01
	2	14	57				
	3	11	87				
	4	0	11				
Vascular invasion	0	31	119	3.56	0.06	0.00	0.99
	1	5	49				
Perineural invasion	0	30	84	13.36	0.00	0.19	0.01
	1	6	84				

### Lgr5 expression at tumor epicenter and invasive front

**Figure 1:**
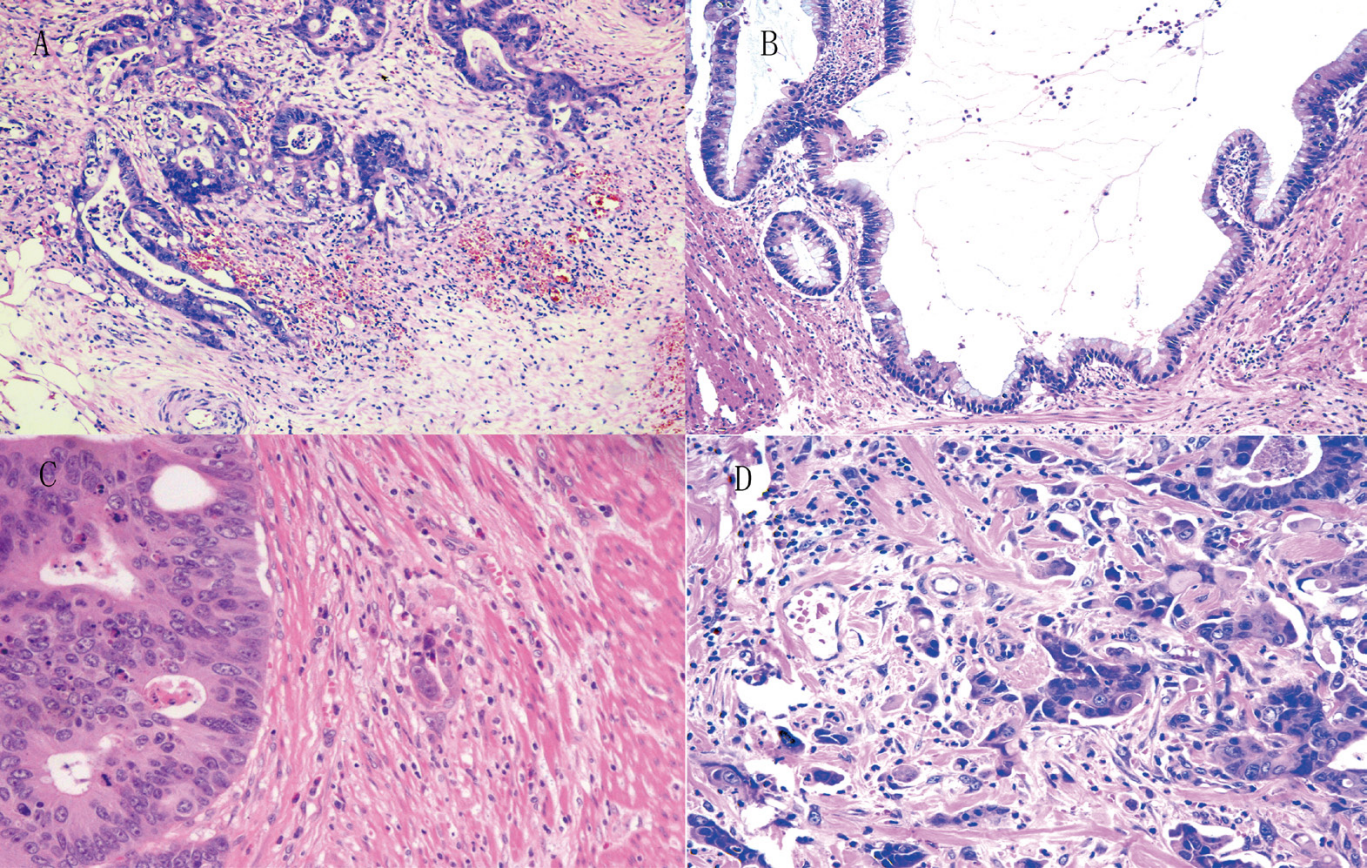
The growth pattern of infiltration (**A**), expanding (**B**) and budding (low grade budding in (**C**) and high grade budding in (**D**)) in CRC.

Of 204 CRC tumors, considerable tumor heterogeneity was observed in tumor border configuration, such as infiltrating and expanding fronts; most tumors showed the phenomenon of TB (Figure 1).

**Figure 2:**
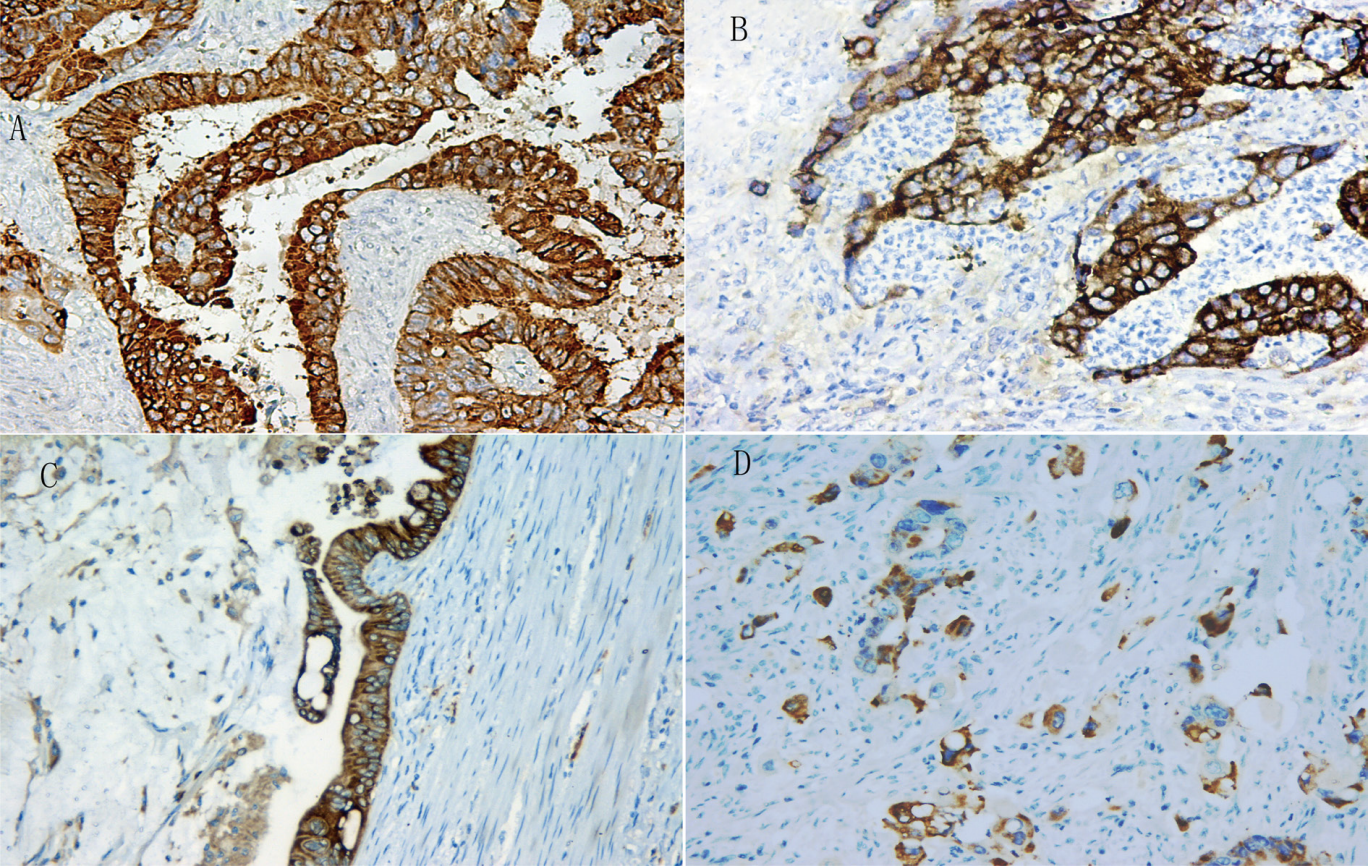
The strong expression pattern of Lgr5 at tumor center (**A**), infiltrating margin (**B**), expanding front (**C**) and tumor budding (**D**) in CRC.

**Figure 3:**
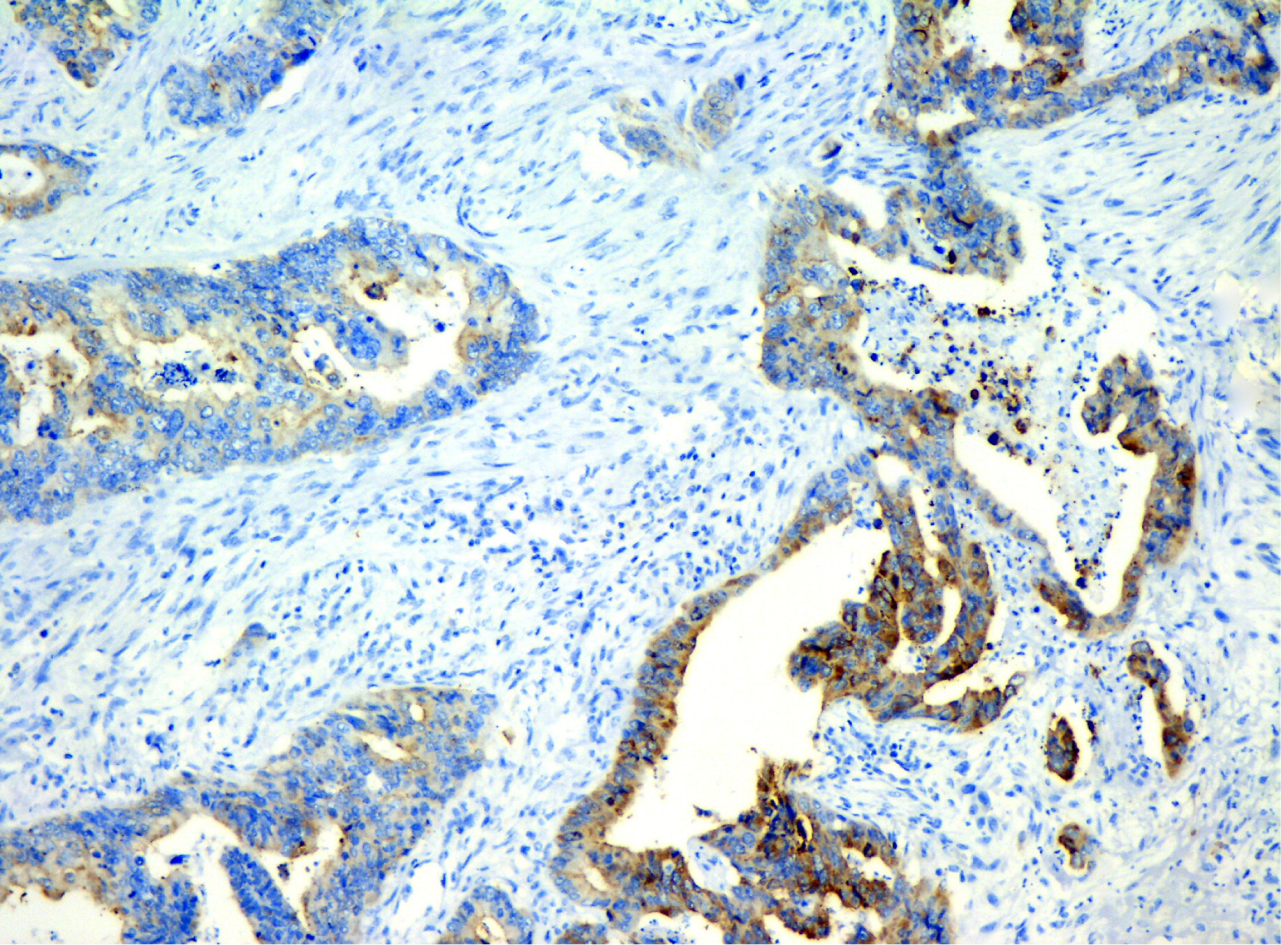
The heterogeneous expression of Lgr5 at at tumor margin and tumor center in CRC

A heterogeneous Lgr5 expression pattern was identified at different tumor sites of CRC (Figure 2). Compared to the tumor epicenter (*n* = 16.7%, 34/204), Lgr5 expression was significantly higher at the infiltrating (*n* = 59.5%, 110/185) and expanding fronts (*n* = 36.4%, 59/162) (*P* < 0.01) (Figure 3). Compared to the expanding front, Lgr5 expression was significantly higher at the infiltrating front (*P* < 0.01) (Table 2).

**Table 2:** Lgr5 expression in infiltrating margin, expanding margin and center of CRC

	*n*	Low (lgr5)	High (lgr5)	*P*
infiltrating margin	185	75	110	0.00^*^
expanding margin	162	103	59	
center	204	170	34	

^*^:^*^P < 0.01 between two groups.

### Lgr5 expression in tumor budding

**Figure 4:**
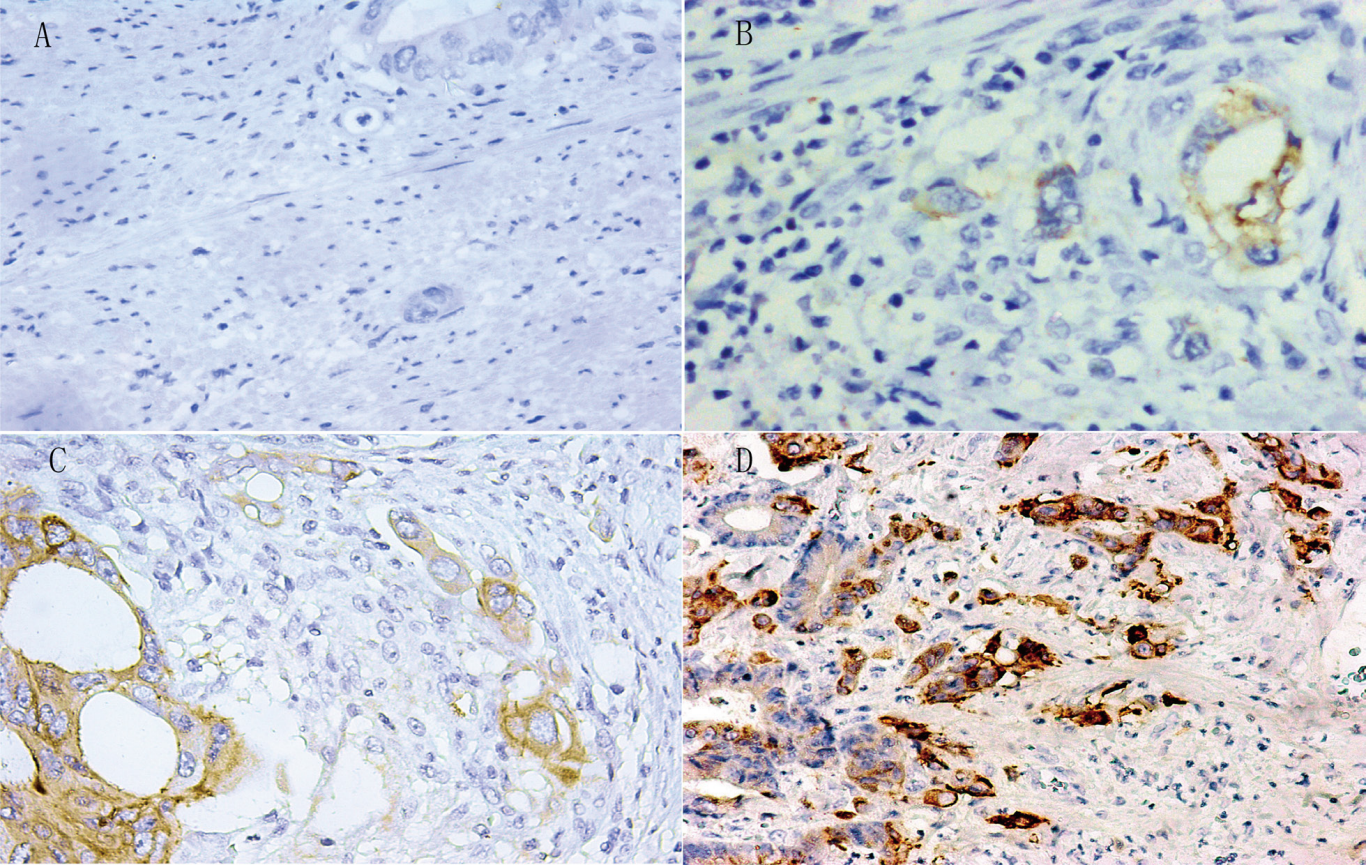
Different expression levels of Lgr5 by immunohistochemistry at tumor budding (negative, weak positive, moderate positive and strong positive staining at TB of CRC in (**A**), (**B**), (**C**) and (**D**) respectively).

Tumor budding (TB) was found in 145 (71.1%, 145/204) tumors, of which 81% (118/145) showed Lgr5 expression (Figure 4). High Lgr5 expression was found in 39.3% (57/145) of TBs and significantly correlated to the TB grade (*r* = 0.19, *P* < 0.05) (Table 3), while a high TB grade was significantly correlated to the depth of invasion, lymph node metastasis, TNM stage, and perineural invasion (*P* < 0.01), but not to patient gender, age, tumor size, tumor location, differentiation and lymphovascular invasion (Table 4).

**Table 3:** Lgr5 expression in TB and its relationship with clinicopathlogical characteristics of CRC

		Lgr5 expression in TB		X^2^	*P*	*r*	*P*
		Low	High				
Gender	Male	48	36	1.05	0.30	−0.05	0.57
	Female	40	21				
Age (years)	≤ 64	45	33	0.64	0.43	−0.05	0.55
	> 64	43	24				
Tumor size (cm) (average)	≤ 4.4	56	33	0.48	0.49	−0.01	0.93
	> 4.4	32	24				
Tumor location	Right-side	25	16	0.00	0.97	0.03	0.76
	Left-side	63	41				
Tumor grade	Low	73	48	0.04	0.84	−0.06	0.48
	High	15	9				
pT	1	1	0	2.03	0.61	0.02	0.80
	2	8	8				
	3	71	42				
	4	8	7				
pN	0	35	24	0.55	0.76	−0.01	0.96
	1	40	27				
	2	13	6				
pM	0	84	52	0.46	0.50	0.08	0.36
	1	4	5				
pTNM	1	5	3	1.55	0.69	0.02	0.78
	2	28	20				
	3	51	29				
	4	4	5				
Vascular invasion	0	59	45	2.42	0.12	−0.17	0.04
	1	29	12				
Perineural invasion	0	49	27	0.96	0.33	0.05	0.57
	1	39	30				
TB Grade	1	42	18	7.18	0.03	0.19	0.02
	2	28	16				
	3	18	23				

**Table 4:** Relationship of TB grade with clinicopathlogical characteristics of CRC

		TB grade		X^2^	*P*	*r*	*P*
		Low	High				
Gender	Male	67	53	0.75	0.39	−0.04	0.62
	Female	52	32				
Age (years)	≤ 64	66	45	0.13	0.72	0.02	0.83
	< 64	53	40				
Tumor size (cm) (average)	≤ 4.4	74	47	0.98	0.32	0.00	0.97
	> 4.4	45	38				
Tumor location	Right-side	33	25	0.07	0.79	−0.04	0.56
	Left-side	86	60				
Tumor grade	Low	104	67	2.69	0.10	0.10	0.15
	High	15	18				
pT	1	6	0	20.55	0.00	0.31	0.00
	2	25	4				
	3	82	68				
	4	6	13				
pN	0	70	28	17.02	0.00	0.26	0.00
	1	42	40				
	2	7	17				
pM	0	115	78	1.45	0.23	0.10	0.14
	1	4	7				
pTNM	1	24	0	24.05	0.00	0.32	0.00
	2	44	27				
	3	47	51				
	4	4	7				
Vascular invasion	0	87	63	0.03	0.87	−0.01	0.85
	1	32	22				
Perineural invasion	0	78	36	10.82	0.00	0.22	0.00
	1	41	49				

## DISCUSSION

Here, immunostaining of Lgr5 was performed in 204 cases of CRC, and the results showed that Lgr5 expression in CRC tumors staged as pTNM I-IV was heterogeneous, but significantly higher at the infiltrating front than at the expanding front, or at the tumor epicenter. High Lgr5 expression in tumor buds was also significantly correlated to high tumor budding, positively correlated to the depth of invasion, lymph node metastasis, pTNM stage, and perineural invasion. The results were consistent with our previous immunohistochemical findings of Lgr5 expression in 42 CRC cases staged as pTNM IV [6]. The present data further confirmed that Lgr5 expression varied at different sites of CRC, which might be ascribed to the intrinsic heterogeneity of CRC and the differences in the invasiveness of cancer cells at different sites. Previous studies showed that CRC tumors with an infiltrating front was associated with a poorer prognosis, as compared to those with an expanding front [7–9]. Our findings in this study suggest that CRC tumors with high Lgr5 expression are more likely to have invasive and metastatic potentials, which remains to be further investigated.

In the present study, our data showed that Lgr5 expression was significantly associated with the depth of tumor invasion, lymph node metastasis, and perineural invasion, parallel to those reported previously in the Chinese CRC patients [10] and also in the Japanese patients [11]. The study results confirmed that Lgr5 expression was significantly correlated to the tumor stage, and suggested that Lgr5 might play an important role in the CRC progression.

Tumor budding reflects a detachment of tumor cells at the invasive front of CRC as single cells or small clusters of neoplastic glands and cells migrating through desmoplastic stroma, demonstrating the characteristics of epithelial-mesenchymal transition and cancer stem cells [12]. Those detached tumor budding cells are more likely to migrate and invade normal tissues, have the anti-apoptotic capability, and express molecules related to a mesenchymal phenotype. Those cells invade surrounding tissues, escape from the host immune responses, metastasize to the distant organs through lymphovascular channels [13, 14]. Our results demonstrated that a high TB grade was positively related to the depth of invasion, lymph node metastasis, TNM stage and perineural invasion, which suggests a higher degree of invasiveness and metastasis of CRC. van Wyk HC et al. also proposed that the number of TB was a predictive factor of lymphovascular invasion and distal metastasis [15].

Recently, some studies indicated that CRC tumor budding cells had high expression of markers of cancer stem cells, such as CD44, CD133 and ABCG5 [16, 17], and suggested that some tumor budding cells in CRC might contain subgroups of cancer stem cells [18]. Lgr5 is one of target genes of theβ-catenin/WNT pathway and the marker of stem cells in small intestine and colon [3] and potential cancer stem-like cells of CRC [4]. The Lgr5 cancer stem cell possibly involves the pathogenesis, progression, and metastasis of CRC, because the expression of Lgr5 was significantly associated with cancer grade, the depth of tumor invasion, perineural invasion, lymph node metastasis, distant metastasis, pTNM stage and Ki-67 expression, as shown in our and other studies [6, 10, 11]. We further showed that high Lgr5 expression in CRC was positively correlated to the TB grade, suggesting the cancer stem cell features of those tumor budding cells in CRC. We speculate that the increase in the stemness of cancer cells in TB of CRC may play a significant role in local invasion and metastasis.

There were some limitations in this study. The tumor size was relatively large in some cases, and entire tumor samples could not be completely examined by histology and immunohistochemistry. In addition, as a retrospective study, patients with stage III CRC accounted for a large proportion and only a few patients were diagnosed as CRC stage I.

Taken together, our results demonstrate that Lgr5 expression in CRC is higher at invasion fronts than at the tumor center in a heterogeneous fashion. At the same time, the results further show that Lgr5 expression in CRC cells are more likely to migrate and invade normal tissues, especially in tumor budding cells, Lgr5 expression cells may have a higher potential to invasion and metastasis. Because Lgr5 is a well-known stem cell marker, Lgr5-expression tumor budding cells at the invasion front may have cancer stem cell characteristics, especially in those high-grade tumor budding cells, which remains to be further investigated.

## MATERIALS AND METHODS

### Samples

CRC tissues were collected from consecutive 204 patients treated at the Nanjing Drum Tower Hospital with radical resection of CRC between May 2014 and September 2015. All resected CRC tumors were routinely processed with a standard anatomic pathology protocol with 4–6 tumor sections taken from each tumor. Tumor histology sections of each case was reviewed and investigated by two experienced pathologists independently. Discrepancy was minimal and resolved with a joint reading to reach a consensus. All tumors were staged (pathologic tumor-node-metastasis stage, pTNM) with the guidelines for CRC of the American Joint Committee on Cancer, 7th edition [19]. Following information was collected and statistically compared: age, gender, tumor location, tumor size, tumor grade, pTNM stage, lymphovascular and perineural invasion. CRC tumor blocks rich in neoplastic cells over 85% of the tumor volume and minimal necrosis were selected for immunohistochemistry study of Lgr5. This study protocol was approved by the Medical Ethics Committee of the Nanjing Drum Tower Hospital.

### Immunohistochemistry

Routine immunohistochemistry was performed in 4-μm sections with the two-step EnVision method. The rabbit anti-human Lgr5/GPR49 polyclonal antibody (Abcam, USA; 1:150) were used with the EnVision immunohistochemistry kit along with the DAB (spell out) solution (Dako, Denmark). In cases with negative Lgr5 immunoreactivity, one additional tumor block from the same case was used to confirm the staining results. Appropriate negative and positive controls were included in each run to validate the immunostaining methods.

Evaluation of Lgr5 expression was carried out with the methods published previously [20, 21]. In brief, the overall staining was the results of the staining intensity and proportion of positive cells. Staining intensity was divided into 4 categories as 0, no staining; 1, light yellow; 2, yellow and 3, brown-yellow. Proportion of positive cells were divided in 5 groups as 0, ≤ 5%; 1, 6–25%; 2, 26–50%; 3, 51–75% and 4, ≥ 76%. The total Lgr5 immunostaining score was the product of staining intensity by the staining score and ranged from 0 to 12: ≤ 1, negative; > 1, positive; > 4, high expression.

### Pathological evaluation of tumor fronts

The “expanding front” was defined as the tumor showed a pushing edge, typically was well circumscribed. In contrast, the “infiltrating front” was defined as the tumor grew in a diffuse, widespread penetration into surrounding soft issues with desmoplastic stroma without a clear border between the tumor and the surrounding tissue, as previously described [22].

### Evaluation of tumor budding

Tumor budding (TB) was defined as a detachment of tumor cells at the invasive front of CRC as single cells or clusters up to five neoplastic cells in the surrounding fibrous stroma. Microscopically, the distribution of cancer cells was inspected at a low view. The region rich in TB was selected for further semi-quantitative evaluation and grading with the methods described previously [20, 21]. Based on the number of TB at a high magnification (×20 objective, 0.95 mm^2^), TB was divided into 4 groups as no TB, grade 0; 1–4 TBs, grade 1; 5–9 TBs, grade 2; ≥ 10 TBs, grade 3. Grade 0–1 refers to low grade, and grade 2–3 to high grade.

### Statistical analysis

Statistical analysis was performed with SPSS version 17.0 (SPSS, Chicago, IL, USA). Comparison between two groups was carried out with the Chi square, Fisher’s exact or Nonparametric tests, when appropriate. Correlation analysis was performed with Spearman rank correlation analysis. A value of *P* < 0.05 was considered statistically significant.
